# Green exercise as a potential pathway to promoting mental health in children and adolescents: a narrative review

**DOI:** 10.3389/fpsyt.2026.1948114

**Published:** 2026-10-16

**Authors:** Huihuang Huang, Xu Peng

**Affiliations:** 1 Department of General Education, Anglo-Chinese College, Fuzhou, China; 2 College of Physical Education and Health Science, Guangxi Science & Technology Normal University, Laibin, China

**Keywords:** adolescents, children, green exercise, mental health, natural environments

## Abstract

Poor mental health during childhood and adolescence may adversely affect emotional regulation, social relationships, education, everyday functioning, and well-being into adulthood. Green exercise—defined as physical activity undertaken with direct exposure to natural environments—has attracted increasing attention as a potentially accessible and low stigma complement to established mental health care. This narrative review synthesized 12 English language empirical studies published since 2000 involving children and adolescents aged 18 years or younger engaging in green exercise. The findings were organized around four mechanism relevant themes: (1) psychological restoration and stress regulation; (2) mastery, autonomy, and person–activity fit; (3) activity engagement and substitution for sedentary behavior; and (4) social and contextual support. Across the evidence base, green exercise was associated with positive affect, life satisfaction, happiness, and self-esteem, as well as, in some contexts, lower levels of stress, anxiety, internalizing symptoms, or psychosomatic symptoms. However, findings varied according to activity type and structure, duration and frequency, participant preferences, sex, and social or environmental context. Brief experiments showed no consistent additional psychological benefit of greener exercise environments, whereas observational and multi day intervention studies suggested possible benefits from sustained participation, manageable challenge, restorative environmental engagement, and supportive contexts. Most evidence was observational or self-reported, nature exposure was inconsistently characterized, and proposed mechanisms were rarely tested prospectively. Cultural meanings of natural environments were also largely unexplored. Consequently, the optimal activity type, intensity, frequency, and environmental conditions remain uncertain. Overall, green exercise appears to be a promising but context dependent complement to child and adolescent mental health promotion rather than a universally effective intervention.

## 1 Introduction

Childhood and adolescence are critical periods of psychological, emotional, and social development. Many mental disorders emerge during adolescence and can have lasting educational, health, and social consequences (1). According to the World Health Organization, approximately one in seven adolescents aged 10–19 years experiences a mental disorder, accounting for 15% of the global burden of disease in this age group (2). Depression, anxiety, and behavioral disorders are among the leading causes of adolescent illness and disability (2). Regional evidence further illustrates the scale of the problem. An analysis of Global Burden of Disease 2021 data from 54 Asia–Pacific countries found that mental disorders were the leading non communicable cause of years lived with disability among aged 0–19 years in 2021, while related disability adjusted life years increased by 10.07% between 1990 and 2021 (3). Child and adolescent mental health problems therefore impose a substantial global and regional burden.

Evidence based psychological treatments are available for mental health problems experienced by children and adolescents (4). Trauma focused cognitive behavioral therapy, for example, alleviates pediatric post-traumatic stress symptoms, with effects stable at 12 month follow up (5). Such treatments represent established clinical care, whereas green exercise is examined here as a complementary environmental behavioral approach. Nevertheless, treatment efficacy alone does not ensure population level impact. Few young people requiring mental health services receive appropriate care, and specialist psychotherapy alone cannot meet population needs (4). Major barriers include shortages and unequal geographical distribution of trained professionals, limited service capacity, poor access, and stigma (1, 4, 6, 7). Green exercise may complement existing services because it can be integrated into everyday life, schools, and community settings and may carry relatively little stigma (8). Green exercise is defined as physical activity undertaken while directly exposed to nature (9–11). Natural environments include green spaces such as parks, forests, and countryside, blue spaces such as rivers and lakes, and other environments containing substantial natural elements (10). Physical activity functions as the broader behavioral category, whereas exercise refers to its planned and structured form (10). Outdoor physical activity and play describe the location or form of activity but do not necessarily establish that the environment is natural or green. These terms are therefore retained when reporting original studies rather than treated as synonyms for green exercise. Because some studies did not fully characterize nature exposure, their settings were interpreted along a continuum from general outdoor to explicitly natural environments, with this variation treated as a source of heterogeneity.

The potential psychological benefits of natural environments are supported by several established theories. Attention Restoration Theory proposes that sustained cognitive demands can deplete directed attention, whereas natural environments may facilitate its recovery through experiences of being away and “soft fascination”, engaging attention without requiring substantial mental effort (12), Stress Reduction Theory emphasizes affective and physiological processes, proposing that safe, non-threatening natural environments may promote positive affect, reduce negative affect and physiological arousal, and facilitate recovery from stress (13). The Biophilia Hypothesis provides a broader evolutionary account of humans’ evolved tendency to attend to and affiliate with living organisms and natural processes, helping explain the psychological salience of nature (14). Together, these perspectives provide complementary explanations for cognitive recovery, affective and stress regulation, and human responsiveness to natural environments. They primarily explain the environmental component of green exercise and therefore directly inform the review’s theme of psychological restoration and stress regulation. However, they do not fully account for benefits arising from physical activity, mastery and autonomy, activity engagement, or social and contextual support. The review therefore also examines these complementary mechanisms and the conditions under which restorative pathways may operate.

Empirical evidence provides preliminary but inconsistent support for the mental health benefits of green exercise. Research involving predominantly adult samples has reported short-term improvements in self-esteem and mood (9). A systematic review similarly identified some psychological benefits of activity undertaken in natural rather than indoor environments, while also emphasizing substantial heterogeneity and poor methodological quality within the evidence base (15). Evidence involving adolescents remains limited and mixed. Among Canadian adolescents, outdoor play was associated with fewer psychosomatic symptoms among girls, whereas greater perceived importance of nature connectedness was associated with a lower prevalence of such symptoms in both sexes (16). During the COVID 19 pandemic, US adolescents who experienced smaller reductions in outdoor activity also reported smaller declines in subjective well-being (17). However, both studies relied on cross sectional self-report data and cannot establish causality.

Experimental evidence also cautions against assuming that exercise in more natural environments is superior to exercise in urban or indoor settings. Running improved schoolchildren’s self-esteem, but the magnitude of improvement did not differ significantly between a rural country park and an urban school campus (18). Another experiment found no consistent nature gradient effect (19). Outcomes may therefore depend on activity type, preferences, autonomy, social context, and the psychological measure assessed, rather than greenness alone.

Several knowledge gaps therefore remain. First, much of the literature on physical activity in natural environments has focused on adults, whereas evidence involving children and adolescents remains limited and heterogeneous (10). Second, green exercise encompasses diverse activity types and settings, complicating studies. Third, many studies cannot separate the effects of physical activity from those from natural environment, social interaction, participant choice, or additional intervention components.

Accordingly, this narrative review synthesizes empirical evidence on green exercise and mental health among children and adolescents. It addresses three questions: (1) What does the existing evidence suggest about the associations between green exercise and psychological distress, positive mental health, and well-being? (2) What restorative, psychological, social, and contextual mechanisms may help explain the reported associations? (3) What methodological limitations characterize the current evidence base, and what priorities should guide future research? Rather than presuming that activity in natural environments is universally superior, the review examines the conditions under which green exercise may—or may not—support the mental health of children and adolescents.

## 2 Methods

A narrative review approach was considered appropriate because the included evidence was heterogeneous in disciplinary perspective, study design, population, exposure, and outcome measures. When statistical pooling is neither feasible nor appropriate, narrative synthesis enables findings from diverse sources to be organized systematically and interpreted thematically. As Grant and Booth noted, review types may differ in the extent of their literature searches and quality appraisal, while their synthesis may take narrative, conceptual, chronological, or thematic forms (20). This narrative review focused on empirical evidence concerning green exercise and psychological outcomes among children and adolescents aged 18 years or younger. It aimed to synthesize reported mental health and well-being outcomes, examine potential restorative, psychological, social, and contextual mechanisms, and identify methodological limitations and priorities for future research. The review procedures comprised database searching, predefined eligibility criteria, structured data extraction, and thematic narrative synthesis. The final database search was conducted on 19 July 2026.

### 2.1 Search strategy and study selection

Literature searches were conducted in PubMed and Web of Science for English language studies published between January 2000 and July 2026. The search strategy combined terms relating to natural environments, physical activity, mental health, and children or adolescents: (“green” OR “natur*” OR “outdoor” OR “outside” OR “park” OR “green space*”) AND (“physical* activ*” OR “exercis*” OR “walk*” OR “cycl*” OR “hik*” OR “leisure time” OR “leisure time” OR “recreation*”) AND (“mental health” OR “mental well-being” OR “psychological well-being” OR “well-being” OR “depression” OR “anxiety” OR “stress” OR “resilience” OR “self-esteem” OR “self-efficacy” OR “stress related disorders”) AND (“adolescen*” OR “child*” OR “youth” OR “young people” OR “teen*” OR “student*” OR “pupil*”).

Studies were included if they: (a) involved children or adolescents aged 18 years or younger, reported a sample mean age of 18 years or younger, or provided separately extractable data for eligible participants within this age range; (b) examined green exercise—conceptually defined as physical activity undertaken with direct exposure to nature—or a closely related outdoor activity exposure, including outdoor physical activity, play, or recreation, as the principal intervention or exposure; because some studies did not fully characterize the natural features of the setting, the degree and type of nature exposure were considered during synthesis rather than assumed to be equivalent across studies; (c) reported at least one psychological outcome, including a measure of mental health, well-being, or resilience; (d) presented original quantitative, qualitative, or mixed methods empirical research; and (e) were published in English in 2000 or later. Because some studies did not fully characterize the natural features of the setting, the degree and type of nature exposure were considered during synthesis rather than assumed to be equivalent across studies.

Studies were excluded if the population or setting was ineligible; physical activity was not a principal exposure or intervention; no psychological outcome was reported; the sample was restricted to a disease specific clinical population without addressing broader child or adolescent mental health concerns; the publication did not present eligible original empirical data; the data duplicated a more complete report; or the full text could not be obtained.

Search results were exported to Endnote 21. Thirty four duplicates were removed using Endnote 21 and a further five were removed manually, leaving 1,832 unique records for title and abstract screening. Potentially eligible articles were assessed in full text against the inclusion and exclusion criteria. Of the 35 articles assessed in full, 12 met the eligibility criteria and were included in the narrative synthesis (**Table 1**). The study selection process is summarized in **Figure 1**.

**Table 1:** Characteristics of studies.

No.	Citation	Study population (N)	Type of green exercise	Key findings	Theme(s)
1	Reed K (2013) (18)	N=75, the UK children aged 11–12	Running: a 1.5 mile timed run in a rural country park versus an urban school campus	Running improved self-esteem overall (F(1,74)=12.2, p<0.001), but improvement did not differ between the green and urban settings (F = 0.13, p=0.72).	T2
2	Hesketh KR & Dodd HF (2026) (21)	N=1,018, the UK preschool children aged 2–4; 47% female	Adventurous play (thrilling play involving risk) and outdoor play, assessed as separate exposures	Adventurous play was associated with lower internalizing scores (β=−0.02) and higher positive affect (β=0.04). Outdoor play was associated with higher positive affect but not internalizing symptoms.	T1, T3
3	Janssen I (2016) (22)	N=20,122, Canadian students in grades 6–10; 47.5% male	Active video games (AVG), sedentary video games (SVG), and active outdoor play (AOP)	Replacing 1 h/day of active outdoor play with active video games was associated with more emotional problems, lower prosocial behaviour, and lower life satisfaction.	T3
4	Zhang J et al. (2021) (23)	N=153 (final sample), Chinese adolescents aged 12–18	Psychological counselling combined with outdoor exercise twice weekly for 8 weeks (50 min/session), including leisure tourism, badminton, basketball, aerobics, rope skipping, and cross country running	Compared with routine education, the combined program reduced anxiety and depression, improved sleep quality, and improved five dimensions of psychological resilience (all p<0.05).	T1, T2, T4
5	Mutz M & Müller J (2016) (24)	Study 1 only: N = 12, German pupils aged 14	Study 1: a 9 day Alpine hiking expedition (~175 km).	Life satisfaction (ES = 0.58) and mindfulness (ES = 1.32) increased, while perceived stress—PSQ Demand—decreased (ES=−0.66).	T1, T2, T4
6	Stagnone N et al. (2025) (25)	3,246 caregivers reporting on US children aged 6–18; activity specific samples: organized sport n=1,258, unstructured PA n=1,220, land use recreation n=1,117	Organized youth sport, unstructured physical activity, and land use recreation (e.g., hiking, paddle boarding, rafting, skiing, mountain biking)	Caregivers perceived greater mental health and emotional control benefits from unstructured physical activity and land use recreation than from organized sport; effects were small. No difference was found for social well-being.	T2, T4
7	Jackson SB et al. (2021) (17)	N=624, US adolescents aged 10–18; quota controlled online convenience sample	Outdoor play and nature based activities	Smaller reported reductions in outdoor play and nature based activities were associated with smaller declines in subjective well-being. The retrospective associations do not establish causality.	T1, T3
8	Frömel K et al. (2017) (26)	N=10,086 for activity preferences; n=2,446 for IPAQ and WHO 5; n=1,278 for pedometer data; Czech and Polish adolescents, mean age approximately 16.7	Preferred outdoor physical activities, including cycling, swimming, skiing, skating, snowboarding, hiking, and horse riding	Adolescents whose preferred activities corresponded with actual participation were more likely to meet activity recommendations and report higher well-being simultaneously (OR = 2.52, 95% CI 1.65–3.83).	T2
9	Piccininni C et al. (2018) (16)	Weighted population N = 29,784; complete case analytic sample n=20,697; Canadian adolescents aged 11–15; 52.7% male	Unstructured outdoor play outside school hours; outdoor locations were not restricted to natural or green environments. Nature connectedness was assessed separately	>0.5 h/week of outdoor play was associated with fewer high psychosomatic symptoms among girls but not boys. Greater perceived importance of nature connectedness was associated with fewer symptoms in both sexes.	T1, T2
10	Wade L et al. (2020) (19)	N=90, Australian adolescents aged 14–15; 57.8% male	The same group based aerobic and body weight resistance circuit conducted under four conditions: indoor, urban park, nature reserve, and no exercise control	Selected differences occurred in sustained attention, cognitive arousal, and vitality, but there was no consistent nature gradient effect across outcomes.	T1, T2
11	Kronaizl SG & Koss KJ (2023) (27)	Longitudinal cohort N = 4,441, US Fragile Families Study; children followed from age 5 to 15; 52.2% male; analytic N varied by outcome	Parent reported total outdoor play hours per week at age 9	More outdoor play at age 9 was associated with greater physical activity and lower anxiety at age 15, but not with BMI or depressive symptoms.	T3, T4
12	Bradbury T et al. (2025) (28)	N=10,413, nationally representative Canadian sample aged 12–17 (2019 CHSCY)	Outdoor physical activity, including biking, skating, gardening, playing ball, and sledding	≥14 h/week was associated with higher odds of positive mental health, life satisfaction, and happiness, independent of indoor physical activity; associations with anxiety, depression, and life stress were not significant.	T1, T3

Theme assignments indicate the review themes to which each study contributed; they do not imply that the study directly tested the corresponding mechanism or theoretical framework. PA, physical activity; AOP, active outdoor play; AVG, active video games; SVG, sedentary video games; OPA, outdoor physical activity; CHSCY, Canadian Health Survey on Children and Youth.

**Figure 1:**
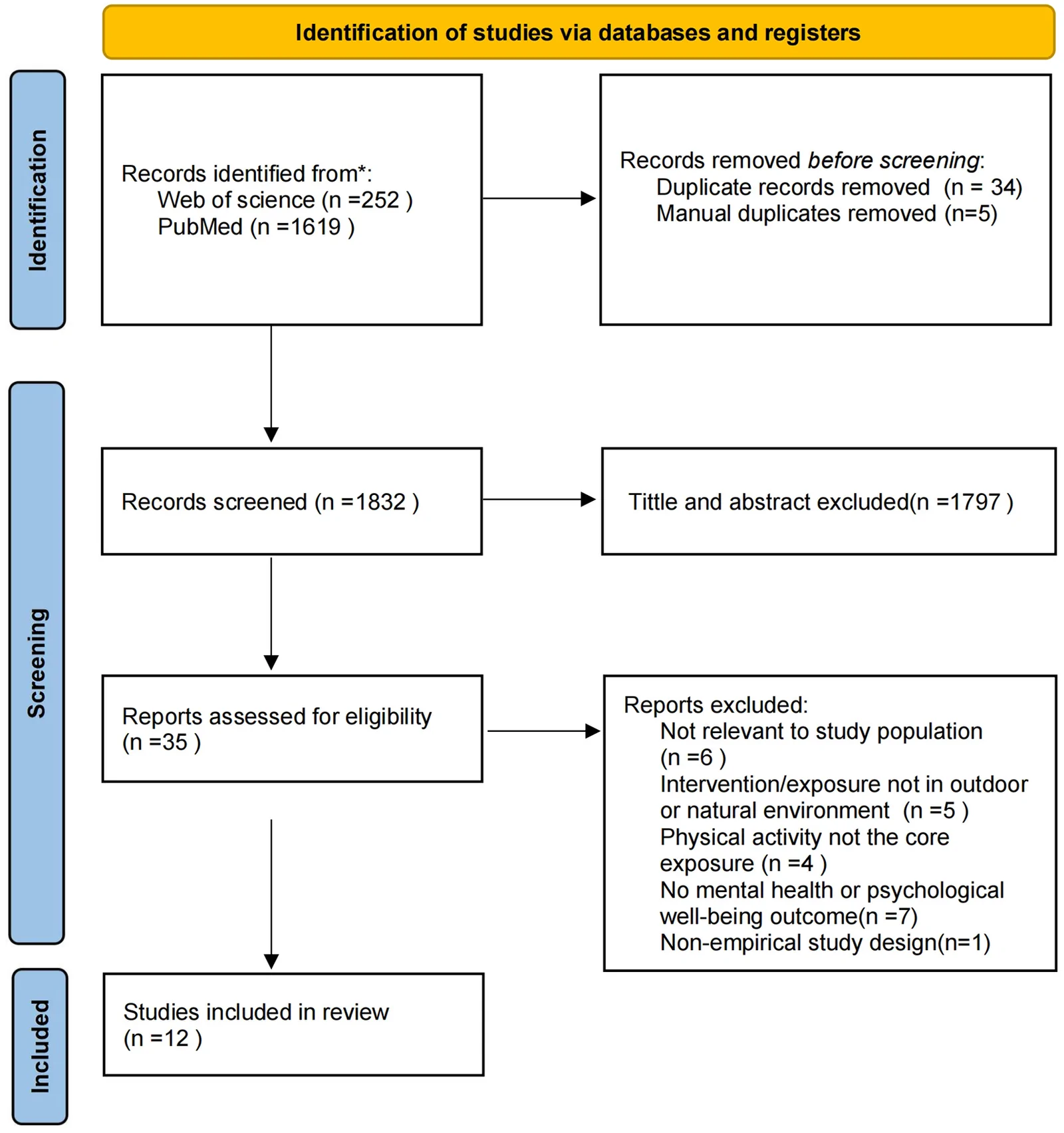
PRISMA flow diagram of the study selection process.

### 2.2 Data extraction and narrative synthesis

For each included study, information was extracted on publication characteristics, country, participant age and sample size, study design, type and setting of green exercise, intervention or exposure duration and frequency, comparison condition where applicable, psychological outcomes, and principal findings.

The themes were developed through an iterative, primarily inductive process rather than being fully established *a priori*. Extracted findings were initially coded descriptively according to the outcomes, proposed mechanisms, activity characteristics, environmental conditions, and contextual factors reported in each study. Codes were then compared across studies and grouped into provisional categories. These categories were iteratively refined with reference to the review aims and the theoretical perspectives introduced in the Introduction, resulting in four mechanism relevant themes: psychological restoration and stress regulation; mastery, autonomy, and person–activity fit; activity engagement and substitution for sedentary behaviour; and social and contextual support. The synthesis integrated findings across heterogeneous study designs and was supplemented with illustrative quantitative estimates where available.

## 3 Results

### 3.1 Characteristics of the included studies

This review included 12 studies conducted in the United Kingdom (UK), Canada, China, Germany, the United States (US), the Czech Republic, Poland, and Australia (16–19, 21–28). Quantitative sample sizes varied considerably, ranging from 12 participants in a pilot study of an Alpine hiking program (24) to 20,122 Canadian students in an isotemporal substitution analysis (22). Participants ranged from preschool children aged 2–4 years (21) to adolescents aged 18 years (17, 23). Of the two studies reported in a single publication, only Study 1, which involved 14 year old students, was eligible for the present review; Study 2 was excluded because its participants were aged 19–25 years (24). This developmental breadth is relevant to the interpretation of the findings because the nature and social context of the activities differed across age groups. The preschool study focused on outdoor and adventurous play (21), whereas studies involving older children and adolescents examined activities such as running, structured outdoor exercise, recreation, nature-based activities, and outdoor adventure programs (17–19, 22–28). Autonomy, caregiver or peer involvement, opportunities for independent activity, and the relevance of particular psychological outcomes may also vary with age. Given the small and heterogeneous evidence base, the findings should not be assumed to apply uniformly across preschool, school-age, and adolescent populations, and age-specific conclusions remain limited.

The evidence base comprised two experimental studies—a repeated measures experiment and a randomized exercise environment trial (18, 19)—one randomized multicomponent intervention (23), one small uncontrolled hiking pilot study (24), six cross sectional surveys (16, 21, 22, 25, 26, 28), one retrospective pandemic period survey (17), and one longitudinal cohort analysis (27). This diversity of study designs offers multidimensional perspectives on the relationship between green exercise and mental health among children and adolescents, but also presents substantial challenges for direct comparison and causal inference.

Following the iterative coding and thematic development process described in the Methods, the findings were organized into four interrelated, mechanism relevant themes: (1) psychological restoration and stress regulation; (2) mastery, autonomy, and person–activity fit; (3) activity engagement and substitution for sedentary behaviour; and (4) social and contextual support. These themes were developed primarily from recurring patterns across the included studies and were subsequently interpreted in relation to the theoretical perspectives outlined in the Introduction. In particular, the first theme was considered in relation to Attention Restoration Theory, Stress Reduction Theory, and the Biophilia Hypothesis, whereas the second theme incorporated a person–activity perspective by examining how psychological outcomes varied according to participants’ preferences, perceived competence, autonomy, and compatibility with particular activities and environments. The final two themes captured behavioral and contextual pathways not fully explained by restoration based theories. The following sections present the findings according to this conceptual organization.

### 3.2 Mechanism related evidence

The included studies primarily reported psychological outcomes rather than formally testing mechanisms of action. None evaluated a complete pathway through which exposure to natural environments influenced mental health via prespecified mediators, such as cognitive restoration, emotion regulation, or social interaction. Accordingly, this review distinguished between: (1) findings from studies that directly measured a proposed mediator and formally tested its indirect statistical effect, classified as mechanism testing evidence; and (2) explanatory interpretations proposed by the study authors on the basis of observed outcome patterns, classified as mechanism hypothesizing evidence. This distinction was used to avoid treating outcome based interpretations as equivalent to empirically tested causal pathways.

#### 3.2.1 Psychological restoration and stress regulation

The most direct evidence of psychological restoration came from a small pilot study of an Alpine hiking program. Among 12–14 year old adolescents who completed a successful nine day hike, scores on the “demand” subscale of the Perceived Stress Questionnaire decreased from 2.38 to 1.93 (effect size [ES] = −0.66, p = 0.022), mindfulness scores increased from 4.41 to 4.81 (ES = 1.32, p = 0.001), and life satisfaction increased from 7.33 to 8.33 (ES = 0.58, p = 0.034) (24). The reported p values were based on one tailed tests. The authors suggested that these changes might be related to the temporary interruption of usual school routines, reduced time and deadline pressure, and greater attention to present moment experiences. These concurrent changes were consistent with reduced perceived demands and enhanced present moment awareness, but did not directly demonstrate attention restoration. Because Study 1 lacked a control group and included no follow up assessment, it was not possible to determine whether the changes resulted from the natural environment or from the interruption of everyday routines, physical exertion, the novelty of the experience, group support, or other contextual factors.

Evidence collected during the COVID 19 pandemic was also consistent with a possible stress buffering pathway. Among US adolescents, smaller declines in participation in outdoor play activities (B = 0.44, p < 0.001) and nature based activities (B = 0.21, p = 0.016) were associated with smaller declines in subjective well-being (17). In addition, 76.4% of adolescents reported that spending time outdoors in nature helped them cope with stress caused by social distancing. These adolescents experienced significantly smaller declines in subjective well-being and self-reported mental health than those who did not report this benefit. However, the pre COVID 19 measures of activity participation and well-being were reported retrospectively, whereas pandemic period measures reflected participants’ current self-reports. The single survey design was therefore susceptible to recall bias and could not establish causality.

Nature connectedness may represent a related affective pathway. In the overall sample of Canadian adolescents, perceiving nature connectedness as “important” was associated with a 25% lower prevalence of high psychosomatic symptoms (adjusted RR = 0.75, 95% CI 0.62–0.91), and this association did not differ significantly by sex or age (16). By contrast, spending at least 0.5 hours per week playing outdoors, compared with no outdoor play, was associated with a lower prevalence of high psychosomatic symptoms only among girls (adjusted RR = 0.76, 95% CI 0.60–0.95). This pattern suggests that the psychological meaning attached to contact with nature may be relevant in addition to the duration of outdoor activity. However, the study analyzed perceived nature connectedness and outdoor play as separate exposure variables and did not test whether either variable mediated the effect of the other or whether they formed a sequential pathway.

Experimental findings imposed important limits on a simple restorative explanation. Wade et al. found that the increase in state level vitality in the park exercise group was greater than that in the non-exercise control group (d = 0.57), although vitality differed between groups at baseline (19). For perceived stress, a moderate effect size favored the indoor exercise group over the park exercise group (d = 0.51), and the study found no conclusive evidence of a nature gradient effect. Reed et al. similarly reported a significant main effect of exercise on self-esteem (F(1, 74) = 12.2, p < 0.001), but no condition by exercise interaction (F(1, 74) = 0.13, p = 0.72), indicating that the pre to post improvement did not differ significantly between the rural country park and urban school campus conditions (18). Taken together, these findings suggest—but do not establish—that the restorative effects associated with natural environments may depend on whether the activity allows sufficient attention to be directed towards the surroundings, rather than arising inevitably from exercising in a greener setting.

#### 3.2.2 Mastery, autonomy, and person–activity fit

Activities involving challenge and choice may influence mental health through perceived competence, autonomy, and emotional learning. Among British preschool children, each additional hour per week spent in adventurous play was associated with a lower internalizing score (B = −0.02, 95% CI −0.03 to −0.01) and a higher positive affect score (B = 0.04, 95% CI 0.03–0.05) (21). The authors proposed that repeated exposure to feelings of excitement and uncertainty may help children moderate their responses to external stimuli that are uncertain or provoke fear. Outdoor play was associated with positive affect but not with internalizing scores, suggesting that adventurous play and outdoor play may have different patterns of association with psychological outcomes. However, the study did not directly compare their effects or test emotion regulation as a mechanism. Moreover, its cross sectional design precluded causal inference and could not rule out reverse causality.

Findings concerning self-perception were mixed. Reed et al. found that running increased self-esteem in both rural and urban environments, but reported no condition by exercise interaction (F(1, 74) = 0.13, p = 0.72) (18). Although the green exercise route was considered more physically demanding because of its rough terrain and changes in elevation, ratings of perceived exertion and enjoyment were similar between the control and green exercise conditions (RPE: 13.6 vs 13.5, p = 0.91; enjoyment: 83.6 vs 89.2, p = 0.66). Energy expenditure was not directly measured. These findings provide only tentative evidence that a more physically demanding green exercise condition may feel as easy and enjoyable as a less demanding urban condition; they do not establish that it is equally acceptable to children at all habitual activity levels. In an adolescent hiking study, self-efficacy did not change significantly despite participants successfully completing the excursion (ES = 0.26, p = 0.188) (24). Mastery therefore represents a plausible interpretation of the effects of adventure based activities, but it has not been directly established as a mediator of the observed psychological outcomes.

Autonomy and activity preferences provide additional indirect evidence. Caregivers attributed greater benefits for children’s mental health and emotional control to unstructured physical activity and land use recreation than to organized youth sport, although the effect sizes were very small (η² = 0.006 for mental health and η² = 0.005 for emotional control) (25). The authors proposed self-determination, independence, and positive social engagement as possible explanations, but these constructs were not directly measured and the interpretation therefore requires further testing. Relatedly, Czech and Polish adolescents whose preferred physical activities corresponded with the activities they actually performed had higher odds of simultaneously meeting physical activity recommendations and reporting higher well-being (adjusted OR = 2.52, 95% CI 1.65–3.83) (26). Although this study focused on outdoor physical activity preferences, the reported odds ratio was based on overall agreement between preferred and actually performed physical activities rather than green exercise specifically. The result is consistent with a person–activity fit framework. However, both studies were cross sectional. Overall, the available evidence indicates an association between activity fit or autonomy and positive psychological indicators, but it cannot establish the direction of causality or determine whether preference itself, independently of actual activity matching, improves well-being.

#### 3.2.3 Activity engagement and substitution for sedentary behaviour

A third possible pathway is that outdoor activity may support mental health by replacing sedentary behaviors, such as screen time, with meaningful physical activity. In a nationally representative Canadian sample, after adjustment for indoor physical activity and other covariates, participation in at least 14 hours of outdoor physical activity (OPA) per week, compared with no OPA, was associated with higher odds of positive mental health (adjusted odds ratio (aOR = 1.64, 95% CI 1.13–2.38), high life satisfaction (aOR = 1.75, 95% CI 1.24–2.46), and high happiness (aOR = 2.36, 95% CI 1.59–3.50) (28). No significant associations were found for anxiety symptoms, depressive symptoms, or life stress. This pattern suggests that OPA may be more consistently associated with positive mental health indicators than with the alleviation of specific symptoms; however, the study did not directly test activity engagement or sedentary behaviour substitution as mechanisms.

Isotemporal substitution modelling further indicated that the type of activity being replaced was important. Replacing one hour per day of active outdoor play (AOP) with one hour per day of active video games (AVGs) was estimated to be associated with a 7% higher probability of high emotional problems, a 3% lower probability of high life satisfaction, and a 6% lower probability of high prosocial behaviour (22). By contrast, replacing sedentary video games (SVGs) with AVGs was associated with more favorable mental health indicators. Because these substitution estimates were derived from cross sectional data, they do not demonstrate that any behavioral substitution actually occurred or establish causality. Rather, the findings are consistent with the interpretation that increasing physical activity or energy expenditure alone may not fully reproduce the experiential and social characteristics of active outdoor play.

Longitudinal evidence suggested a possible developmental cascade. More outdoor play at age 9 was associated with greater physical activity and lower anxiety symptoms at age 15. Outdoor play also partially mediated the association between perceived neighborhood collective efficacy at age 5 and subsequent anxiety symptoms (indirect effect B = −0.003, 95% CI −0.007 to −0.001) (27). No corresponding indirect effect was identified for depressive symptoms (indirect effect B = −0.002, 95% CI −0.005 to 0.001). This was the only included study to use mediation analysis to examine an indirect pathway. Its findings support outdoor play as a behavioral mediator through which neighborhood social conditions may be associated with later mental health; however, outdoor play was not a direct measure of physical activity, and the analysis did not test the psychological effects of exposure to natural environments.

Zhang et al. reported that a multicomponent intervention combining the research based psychological counseling model with outdoor exercise improved anxiety, depression, sleep quality, and psychological resilience among adolescents with anxiety symptoms (23). However, the study did not include either a counseling only group or an outdoor exercise only group. It was therefore impossible to determine whether activity engagement, exposure to the natural environment, or psychological counseling constituted the active intervention component. The study consequently provides evidence concerning the outcomes of a multi-component intervention rather than evidence of a specific mechanism.

#### 3.2.4 social and contextual support

The social environment appears to operate primarily by facilitating or constraining engagement in green exercise, rather than as a consistently demonstrated mediator. In the Fragile Families cohort study, higher perceived neighborhood collective efficacy predicted greater participation in outdoor play, which in turn predicted lower subsequent anxiety (27). This finding supports the interpretation that neighborhood trust and cohesion, together with residents’ willingness to intervene, supervise, and assist children, may create conditions conducive to children’s participation in outdoor activities. However, the study did not directly test whether social interaction during the activity itself produced the psychological outcomes.

Other findings suggest that the social context does not invariably confer psychological benefits. During the COVID 19 restrictions, smaller reductions in outdoor play activities (B = 0.44, p<0.001) and nature based activities (B = 0.21, p=0.016) were more strongly associated with smaller declines in subjective well-being than changes in outdoor family activities, for which the association was not statistically significant (B = 0.06, p=0.128) (17). The authors speculated that prolonged confinement with family members during the pandemic may have increased family stress and thereby diminished the potential benefits of outdoor family activities, although this explanation was not directly tested. In a caregiver survey, organized youth sport, unstructured physical activity, and land use recreation did not differ significantly in caregiver rated social well-being benefits (p=0.2928). Unstructured physical activity and land use recreation received higher ratings than organized youth sport for perceived benefits to mental health and emotional control, although the effect sizes were small (25). Taken together, social contact alone cannot fully explain differences among activity types; autonomy, activity structure, participant preferences, and the quality of interpersonal relationships may be more important than the mere presence of other people.

Across the four themes, the most plausible integrative framework can be described as sequential and conditional. Supportive social contexts, such as neighborhood cohesion and family support, together with physical environments that provide access to natural spaces, may facilitate participation. Engagement in the activity may increase physical activity and displace sedentary behaviour. Challenge, choice, and successful participation may influence emotional states and self-perceptions, as suggested by the associations of adventurous play with lower internalizing scores and of agreement between preferred and actually performed physical activities with higher well-being. Natural environments may also provide restorative benefits when the activity leaves sufficient attentional capacity for participants to engage with their surroundings, as illustrated by increased mindfulness; however, such effects are not inevitable and may depend on whether attention is engaged by the environment. These components are likely to operate in combination, but the available studies have not isolated their respective contributions. Overall, the evidence suggests that green exercise does not operate through a single, clearly established mechanism, but through several plausible and potentially interacting pathways.

## 4 Discussion

### 4.1 Principal interpretation

The 12 included studies provide cautious support for green exercise as a context dependent correlate of, or influence on, mental health, but they do not establish a universal mechanism. The most direct evidence concerns short term changes in affective or stress related states and sustained engagement in physical activity. Evidence concerning mastery, autonomy, and social pathways is suggestive but derives primarily from cross sectional associations, caregiver perceptions, or authors’ interpretations, rather than from formally tested mediation effects.

Across the included studies, associations appeared more consistent for positive mental health indicators, including happiness, life satisfaction, positive affect, vitality, and general well-being, than for reductions in specific symptoms of psychological distress. For example, outdoor physical activity was associated with positive mental health, life satisfaction, and happiness, but not with anxiety symptoms, depressive symptoms, or life stress (28), while outdoor play was associated with positive affect but not with internalizing symptoms (21). However, this pattern was not uniform: longitudinal evidence indicated an association with lower subsequent anxiety but not depressive symptoms (27), and a multicomponent intervention reported improvements in both anxiety and depression (23). Green exercise may therefore be more consistently associated with positive well-being than with symptom reduction, although differences in study design and outcome measurement preclude firm comparative conclusions.

Young people’s engagement in green exercise appears to be shaped by influences at multiple levels. Social and neighborhood contexts may facilitate or constrain opportunities for participation (17, 27), while activity preferences, autonomy, and exposure to manageable challenges may influence continued participation (21, 25, 26). The activity provides physical movement and may displace sedentary behaviour (22, 28). When sufficient capacity remains, nature exposure may also contribute to restoration or changes in stress related and affective outcomes (16, 19, 24). Because these pathways were not consistently tested, outcomes likely reflect interactions among environmental characteristics, activity type, individual experience, and social context, rather than the presence of green space or any single form of activity alone.

The theoretical perspectives introduced earlier help to interpret positive and null findings, but the evidence offers only indirect and conditional support. Attention Restoration Theory predicts greater benefit when an activity allows “being away” and soft fascination, leaving sufficient attention for the surroundings (12). This is more compatible with Alpine hiking (24) than with timed running or station based circuits that direct attention towards the task or other participants (18, 19). Stress Reduction Theory suggests that benefits depend on settings being safe and non-threatening and on participants having sufficient initial stress or negative affect to recover from (13); vegetation alone therefore does not guarantee stress reduction. The Biophilia Hypothesis explains broader responsiveness to living systems but does not imply that all natural environments are equally familiar, meaningful, safe, or restorative (14). Thus, the findings are compatible with restoration and stress regulation under particular conditions but neither directly test these pathways nor demonstrate a universal environmental advantage.

Experimental findings reinforce this interpretation. Running produced improvements in self-esteem in both the country park and urban school campus conditions (18), while another experiment showed no consistent gradient favoring more natural environments across stress, mood state, arousal, vitality, and cognitive performance (19). Although not excluding environmental benefits, these findings suggest that exercise intensity, attentional demands, group structure, activity duration, and the psychological outcome selected may influence whether an additional environmental effect is observed. Claims of synergy between physical activity and natural environment exposure must therefore remain conditional. Only one study formally tested an indirect pathway: outdoor play partly linked earlier perceived neighborhood collective efficacy with subsequent anxiety (27). However, this model concerned social facilitation of outdoor play rather than a mechanism through which green exercise produces psychological change; the other proposed pathways therefore remain untested.

### 4.2 Differences by activity type, dose, and context

Psychological outcomes varied by activity type. Adventurous play was associated with lower internalizing scores and higher positive affect, whereas outdoor play was associated only with higher positive affect and not with internalizing scores (21). However, adventurous play was measured across all locations rather than only outdoor or natural settings. Compared with organized youth sport, unstructured physical activity and land use recreation received more favorable caregiver ratings for mental health and emotional control, although the differences were small (25). A multi day hiking program was accompanied by perceived demand, increased mindfulness, and higher life satisfaction (24), whereas brief running and circuit training experiments did not demonstrate a consistent additional benefit of greener exercise environments (18, 19). Thus, challenge, autonomy, environmental attention, and activity structure may be as important as activity labels.

Exercise intensity and attentional demands may partly explain these differences. A timed 1.5 mile run lasted approximately 10–20 minutes (18), whereas a 21 minute group circuit involved moderate to vigorous intensity (19). The circuit’s station based and social demands may have reduced the “soft fascination” afforded by the environment; timed running may impose similar constraints, although attention to the setting was not measured (18, 19). By contrast, the nine day “Crossing the Alps” program involved prolonged separation from everyday routines and repeated engagement with the natural environment (24). The evidence cannot distinguish the effects of cumulative nature exposure, prolonged physical activity, environmental novelty, group cohesion, and successful completion of the challenge.

Dose was operationalized inconsistently. Each additional hour per week was associated with a slightly lower internalizing score and higher positive affect (21), while girls who reported more than 0.5 hours of outdoor play per week had fewer psychosomatic symptoms than those reporting none (16). At least 14 hours of outdoor physical activity per week showed the strongest positive associations with positive mental health, high life satisfaction, and high happiness. The pattern suggested a possible graded association for life satisfaction and happiness, but not with anxiety, depression, or life stress (28). However, the 0.5 hour threshold was an analytical cut off, only 3% of the sample reported at least 14 hours per week, and both studies were cross-sectional. These studies therefore suggest possible dose-related associations for selected positive well-being outcomes, but they do not establish causality, a minimum effective dose, or greater benefits from longer exposure.

Frequency and continuity may matter beyond cumulative duration. During the COVID 19 pandemic, maintaining participation in outdoor play and nature based activities was associated with smaller declines in subjective well-being (17), while correspondence between preferred and performed activities was associated with meeting activity recommendations and higher well-being (26). These findings suggest that continuity and person–activity fit may support engagement, but adherence was not measured and no study experimentally compared participation schedules.

Studies ranged from preschool play to adolescent recreation and an Alpine expedition, involving different developmental demands and levels of choice (17, 18, 21, 24–28). Family and neighborhood conditions were examined inconsistently but may also moderate outcomes.

Person–activity fit should also incorporate environmental and cultural meaning because natural settings may reflect identity, spirituality, collective memory, access, exclusion, and relationships with place. Among some Indigenous youth, land and nature may support calmness, hope, cultural identity, intergenerational learning, and community relationships (29, 30), but these experiences must be understood alongside colonization, dispossession, cultural disruption, and intergenerational trauma (31, 32). A setting described as green may therefore be culturally sustaining, unfamiliar, inaccessible, or unsafe. As none of the included studies examined these processes, they remain an evidence gap rather than an established mechanism.

### 4.3 Mechanism specific limitations and future directions

The principal mechanistic limitation is that the proposed mediators were rarely quantified. Variables such as mindfulness, perceived stress, vitality, enjoyment, self-efficacy, nature connectedness, and social well-being were generally analyzed as outcomes or parallel correlates rather than temporally ordered mediation models (16, 18, 19, 24, 25). Only one study formally tested an indirect pathway, linking neighborhood collective efficacy to subsequent anxiety through outdoor play rather than a mechanism specific to nature exposure (27). Consequently, this review can identify plausible pathways but cannot determine their relative causal importance.

Future studies should prespecify mechanisms and design measurement accordingly. Tests of the restoration hypothesis require repeated measurements of attention, affective states, and stress at multiple time points (19, 24). Tests of mastery and autonomy should directly assess perceived competence, manageable challenge, choice, self-efficacy, and emotion regulation rather than inferring these processes from participation (21, 24, 25). Tests of activity engagement and sedentary behaviour substitution require objective measurement of physical activity and the sedentary behaviour being displaced (22, 26, 28). Tests of social pathways should assess interactions, perceived support, and group context (17, 25, 27).

Factorial and multi arm trials should separate physical activity from nature exposure by comparing activity and rest across natural and built or indoor environments (18, 19). When outdoor exercise is combined with another intervention, as in the eight week multicomponent program (23), separate intervention arms or process measures are required to identify each component. Repeated assessment of mediators and longer follow up would improve temporal inference in acute and uncontrolled studies (18, 19, 24). Where random allocation is not feasible, prospective cohort studies should measure baseline mental health, prior activity, nature preferences, neighborhood conditions, and time varying covariates.

Dose–response studies should report session duration, frequency, intensity, cumulative exposure, and environmental characteristics because these dimensions were inconsistently measured across studies (16–19, 21–24, 26, 28). Acute responses following 10–21 minute exercise bouts (18, 19) should be distinguished from changes across an eight week multicomponent intervention or a nine day Alpine hiking program (23, 24). Comparative studies should hold physical activity dose constant across environments and measure attentional engagement, enabling total exposure, repetition, threshold effects, and activity–environment compatibility to be distinguished.

Adequately powered studies should conduct planned moderator analyses by activity type, developmental stage, sex, baseline mental health, habitual activity, nature connectedness, individual preferences, and neighborhood context (16, 18, 21, 25–28). Given the mixed findings for psychological symptoms, well-being, and environmental comparisons (16, 18, 19, 21, 27, 28), null findings should be reported alongside positive findings. Qualitative or mixed methods research could further explain these differences and address gaps in the predominantly quantitative evidence.

Research involving communities affected by historical or intergenerational trauma requires a cultural and trauma informed approach. Studies should be co designed with communities and assess culturally grounded outcomes such as identity, belonging, collective efficacy, connection to land, intergenerational relationships, and cultural continuity (29–32). Environmental exposure should be characterized by cultural meaning, governance, accessibility, perceived safety, and histories of displacement or exclusion, rather than physical greenness alone. Program delivery should preserve choice, control, and the right to decline participation and, where appropriate, involve community leaders, Elders, or knowledge holders. This would avoid treating historical trauma solely as an individual deficit and enable cultural reconnection, collective identity, and intergenerational learning to be examined as potential pathways. These recommendations address a gap among the 12 included studies; they should not be interpreted as effects demonstrated by the present evidence base.

### 4.4 Implications for research and practice

The available evidence supports offering varied green exercise options rather than prescribing a single activity or intensity. Adventurous and unstructured activities may provide opportunities for autonomy and manageable challenge, although these mechanisms were not directly measured. Walking or hiking may permit greater engagement with natural surroundings, while park based exercise may be practicable, although its accessibility was not evaluated. Participant choice may also support engagement, but sustained participation was untested. Programs should therefore be reflect intended psychological mechanism and participants’ needs rather than by the assume that greater greenness produces larger effects.

Practice recommendations should remain proportionate to the evidence from the 12 studies. Green exercise may complement broader approaches to promoting child and adolescent mental health when participation is enjoyable, feasible, and aligned with young people’s preferences (18, 21, 26). However, it should not replace indicated psychological or psychiatric care; the only study combining counselling with outdoor exercise did not isolate the exercise effect (23). Implementation studies should document reach, adherence, exercise intensity, environmental exposure, participant preferences, adverse experiences, and access to suitable settings (17, 25, 27).

Cultural responsiveness should be embedded in intervention design, particularly where relationships with land have been disrupted by colonization, displacement, environmental change, or intergenerational trauma. A natural setting is not necessarily culturally meaningful or psychologically safe, whereas community led land based activities may support cultural continuity, shared identity, intergenerational relationships, and collective agency (29, 30). Program content, place, leadership, and evaluation should therefore be determined with communities rather than derived from universalized restoration models.

### 4.5 Strengths and limitations

This review used explicit eligibility criteria for population, exposure, outcomes, study design, language, and publication period. Null and mixed findings were retained, measured evidence was distinguished from hypothesized mechanisms, and multicomponent interventions were not attributed solely to green exercise. Structured searching, predefined eligibility criteria, and explicit synthesis procedures improved transparency, while organizing evidence around potential pathways enabled supportive and contradictory findings to be compared across mental health outcomes.

The 12 included studies nevertheless have several limitations. Most were cross sectional or observational (16, 17, 21, 22, 25–28), and several relied on retrospective or participant and caregiver reported exposures and outcomes (16, 17, 21, 25, 27, 28). Experimental studies had small to moderate samples, acute or uncontrolled designs, or multiple intervention components (18, 19, 23, 24). Mechanisms were rarely prespecified or prospectively tested, leaving reverse causation, residual confounding, and pathway overlap unresolved.

Exposure, dose, outcomes, and assessment timing were highly heterogeneous. Studies examined outdoor or adventurous play, preferred activities, nature based recreation, running, circuit exercise, counselling plus exercise, and hiking, with inconsistent measures of duration, frequency, greenness, intensity, environmental attention, and social interaction (16–19, 21–28). This heterogeneity limited synthesis, and immediate changes cannot be assumed to persist or to represent reductions in clinical symptoms.

Outdoor play and green exercise also overlap only partially. Green exercise entails physical activity with direct nature exposure, whereas outdoor play does not necessarily establish a natural setting or meaningful physical exertion. Including these studies broadened coverage of relevant everyday activities but increased conceptual heterogeneity and the risk of exposure misclassification. Their findings therefore cannot automatically be attributed to nature or interpreted as distinct green exercise effects. Original terminology was retained, and environmental variation was treated as heterogeneity.

The narrative design enabled theoretical interpretation across diverse evidence but required more author judgement than a systematic review, increasing possible selection and interpretive bias (20). Although structured procedures improved transparency, the search covered only PubMed and Web of Science, was limited to English language studies published from 2000, and included no formal quality or risk of bias appraisal. The themes should therefore be considered an organizing framework, not a validated causal model or graded statement of evidence certainty. Future systematic reviews should broaden searches, formally appraise bias and certainty, and examine sensitivity to study quality and verified nature exposure.

Finally, the evidence was geographically concentrated in high-income settings in Europe, North America, and Australia, together with one study from China. Most participants were recruited from community or school populations, and only one study specifically involved adolescents with anxiety symptoms (23). These characteristics limit the generalizability of the findings to other cultural contexts, clinical populations, and resource-constrained settings.

## 5 Conclusion

Green exercise was associated with positive affect, life satisfaction, happiness, and self-esteem and, in some studies, with lower perceived stress, anxiety, internalizing symptoms, or psychosomatic symptoms among children and adolescents aged 18 years or younger. The 12 included studies support four potentially interacting mechanism relevant pathways: psychological restoration and stress regulation; mastery, autonomy, and person–activity fit; activity engagement and substitution for sedentary behaviour; and social and contextual support. The most direct evidence concerned short term restorative or stress related changes and activity engagement, whereas evidence for mastery and social pathways was largely indirect or inferential.

Experimental studies did not demonstrate that greener environments were consistently superior to urban or indoor exercise settings, and no study tested a complete causal mediation model linking nature exposure to mental health outcomes. Green exercise should therefore be regarded as a promising but context dependent health promotion strategy whose outcomes may arise from interactions among physical activity, environmental characteristics, individual experience, and social context. Future research requires controlled longitudinal and factorial studies that prespecify and repeatedly measure potential mediators to determine how green exercise may operate, for whom it may be beneficial, and under what conditions.
